# The impact of continuous use intention of cooperative members on new agricultural technologies

**DOI:** 10.3389/fpsyg.2023.1089362

**Published:** 2023-02-22

**Authors:** Fang Jia, Runhan Zhang, Jiajun Li

**Affiliations:** ^1^School of Management, Northwestern Polytechnical University, Xi’an, China; ^2^Department of Justice, Shaanxi Police College, Xi’an, China

**Keywords:** farmers’ cooperatives, new agricultural technology, unified theory of acceptance and use of technology, continuous use intention, social impact of cooperatives, policy support

## Abstract

The widespread application of new agricultural technologies promotes an increase in agricultural production and income and in the optimization and adjustment of the industrial structure. However, there are problems such as low promotion efficiency, an insufficient transformation of achievements, and a mismatch of supply and demand in the process of promotion. Based on the research context of farmer cooperatives in China, this study explores the factors influencing the continuous use intention of cooperative members toward new agricultural technologies and builds a research structure based on the unified theory of acceptance and use of technology (UTAUT) model, which includes performance expectations, effort expectations, cooperative social impact, and policy support. A total of 401 valid questionnaires were collected, and the data were analyzed in three stages using descriptive statistics, a measurement validation model, and a structural equation model, using a survey questionnaire and by inviting members of farmer cooperatives within China to participate in an online survey through a web-based electronic questionnaire. The results of the study found that policy support plays a dominant role in the intention of cooperative members of farmers to use new agricultural technologies consistently, and cooperative social impact plays a facilitating role, while factors such as performance expectation and effort expectation also have a significantly positive effect on the intention to use consistently.

## 1. Introduction

China is a large agricultural country. Agricultural development is the fundamental basis for national stability and sustainable social development. Therefore, the Chinese government pays great attention to three rural issues. Agriculture, rural areas, and farmers are three issues related to rural development in mainland China. The implementation of the agricultural revitalization strategy would promote the modernization of agriculture and improve the sense of access, happiness, and security of farmers. According to the endogenous growth theory, the progress of agricultural science and technology is at the core of the development of modern agriculture and the construction of agricultural modernization (Mao et al., 2013). The Central Committee of the Chinese Communist Party has issued a series of “Central No. 1” documents. The focus is on agricultural science and technology. The basic national policy of agricultural development is to continuously increase the progress and innovation of agricultural science and technology. It includes the promotion of new technologies such as biotechnology, information technology, gene technology, and genetic breeding. The wide application in agricultural production has greatly promoted the development of agriculture in terms of intelligence and information technology.

According to the classification standard of the World Bank, small farmers are defined as those with an arable land area of 30 acres or less. Based on the data of the third agricultural census, small farmers account for more than 98% of the main agricultural operators in China. Small farmers account for 90% of the agricultural workforce. The area of arable land operated by small farmers accounts for 70% of the total arable land area. Therefore, China's “big country and small farmers” remains the long-term basic national condition (Lu and Kong, 2019). It severely restricts the synergistic development of individual farmer production and mechanized operation, industrial development, and technical quality improvement. This has led to diminishing the marginal efficiency of production, weakened risk resistance, and reduced returns. Therefore, the contradiction between “small production” and “big market” is a common phenomenon (He, 2019). This leads to information asymmetry, homogeneous competition among farmers, weak sustainability of agricultural production, and disproportionate inputs and outputs.

Farmer cooperatives are an effective vehicle for developing moderate-scale agricultural operations and promoting agricultural development. They are also an important lever for the progress of agricultural science and technology. According to the statistics of the Ministry of Agriculture, by the end of April 2021, the number of farmer cooperatives registered nationwide reached 2.259 million, with more than 10,000 joint cooperatives, driving nearly 50% of farm households in China. Therefore, as the basis of rural revitalization in China and as an effective carrier of innovative rural science and technology service systems, the continued use of new agricultural technology by farmer cooperatives will certainly drive the promotion and application of certain technologies (Wang et al., 2020). In the process of promoting the application of new agricultural technologies, there still exists a shortage of funds for cooperatives, and the scale of operations is too small. This inhibits the demand for new agricultural technologies. The cognitive barriers and information asymmetry of farmers hinder the application of new agricultural technologies and cause other problems, resulting in a low adoption rate of new agricultural technologies.

At the same time, some scholars conducted studies based on the intention of individual farmers to use new agricultural technologies consistently. For example, Han et al. (2022) found that perceived usefulness, perceived ease of use, and external environment had significant effects on the continuous use intention of farmers toward the plant protection drones, and the literacy level of farmers played a moderating role in the continuous use intention toward the drones. Gao and Gu (2021) found that information quality has a significant impact on the intention to continuously use the WeChat agricultural science and technology public account, while service quality had an indirect impact on the intention to continuously use through satisfaction. Finally, in exploring the factors influencing the behavior of sustained use of soil testing by farmers of different scales, Li et al. (2018) found that sustained use behavior was directly influenced by the difference in scale, i.e., the highest percentage of use by farmers in the large-scale group. However, most of the existing studies on the success of information systems have focused on user perceptions and neglected to consider the factors influencing the intention of cooperative members to use new agricultural technologies consistently in the context of farmer cooperatives.

Therefore, this study examined the intention of Chinese farmer cooperative members to use new agricultural technologies consistently and the related influencing factors. Using the UTAUT model as a framework, the relationship between performance expectations, effort expectations, cooperative social impact, policy support, and continuous use intention is empirically demonstrated. The purpose of this study is to understand the factors that influence the intention to continuously use new agricultural technologies and to make relevant recommendations to government departments and farmer cooperatives in order to accelerate the application of new agricultural technologies in agricultural production.

## 2. Literature review

### 2.1. Farmer cooperatives

According to the Law of the People's Republic of China on Professional Farmers' Cooperatives issued in 2017, farmer cooperatives are based on the rural household joint production responsibility system: agricultural production operators or providers and utilizers of agricultural production and management services, voluntary association, and democratic management of mutual economic organizations. With the rapid development of farmer cooperatives, cooperatives play a “bridge” role in the process of agricultural science and technology promotion, effectively making up for the lack of supply of the agricultural science and technology promotion system of the government. In addition, cooperatives play a “leveraging role” between agricultural research institutes, public welfare extension agencies, and farmers, which is conducive to the balance between new agricultural technology research and the needs of farmers, thus promoting the application of new agricultural technologies (Yang and Li, 2020). The advantages of agricultural cooperatives are mainly reflected in (i) the purpose of serving cooperative members, which ensures that agricultural science and technology promotion is oriented to the needs of farmers; (ii) the membership system in which owners, promoters, and users are united, which ensures an effective match between agricultural science and technology promotion and the needs of farmers; and (iii) the service function of the unified purchase of agricultural materials and the unified sale of agricultural products, which effectively reduces the risk of new technologies (Yuan, 2017).

Therefore, farmer cooperatives in this study are organizations that are formed based on joint family production contracting, have the nature of economic entities, and are engaged in business activities such as production, processing, and marketing in the agricultural field.

### 2.2. Performance expectations, effort expectations, and continuous use intention

In the UTAUT model, performance expectation is the degree to which an individual believes that using a system or technology will help them improve job performance. Effort expectation is the level of effort an individual is expected to be willing to exert when using a particular technique (Venkatesh et al., 2003). Continued use intention is the willingness of users to continue to use an information system after they have experienced it (Bhattacherjee, 2001). The research on the continued use intention is broadly based on the TAM model (Min et al., 2019), the ECM-ISC model (Tam et al., 2020), the D&M model (Shim and Jo, 2020), and the UTAUT model (Yuan et al., 2015). Scholars achieve rich results. Therefore, in this study, the continued use intention refers to the subjective willingness of members of farmer cooperatives to continue using the new agricultural technology after the initial use of the technology.

The UTAUT model has been commonly applied in diverse domains such as online learning (Li and Zhao, 2021), social media (Gruzd et al., 2012), e-government (Liang et al., 2015), and mobile Internet (Jiang et al., 2020) to understand factors related to the behavioral intention of users to use technology. In terms of empirical research on performance expectations on continuous use intention, Shen (2018) constructed a model of factors influencing the continuous use intention of VR video users based on the UTAUT model when studying the factors influencing the continuous use intention of VR video users combined with the functional characteristics of VR video. The empirical analysis found that performance expectations significantly and positively affect the continuous use intention of VR video users. While studying the factors influencing the continuous use intention toward mobile news apps in India, Cheng et al. (2020) found that performance expectations significantly and positively influence the continuous use intention of users. Finally, Tam et al. (2020), in their study of the factors influencing the intention to keep using mobile applications, constructed a model of the influence of the intention to keep using mobile applications based on the ECM model and the UTAUT2 model. They found through empirical analysis that performance expectations positively and significantly influence continued use intention.

In terms of empirical research on effort expectations on continuous use intention, Li and Zhao (2021) found through empirical analysis that effort expectations significantly and positively influenced the continuous use intention of students toward massive open online courses (MOOC). Liang et al. (2015), while studying the factors influencing the intention of the public to continuously use the online government office hall, fused the UTAUT model with trust theory to construct a research model of the intention of the public to continuously use the online office hall in Guangdong Province. They found through empirical analysis that effort expectations significantly and positively influenced the intention of the public to continuously use the online office hall. Finally, Gao (2012) explored the factors influencing the intention of college teachers to use online teaching methods consistently based on the UTAUT model. The study showed that effort expectations significantly and positively influenced the continued use intention of college teachers toward online teaching methods.

Based on this, we propose the following hypotheses:

H1: Performance expectations will significantly and positively impact the continuous use intention toward new technologies in agriculture.
H2: Effort expectations will significantly and positively impact the continuous use intention toward new technologies in agriculture.

### 2.3. Cooperative social impact and continuous use intention

Social impact (SI), which belongs to the category of psychology, refers to the social interactions of an individual in which he or she is influenced by others or groups, leading to a change in the attitudes, beliefs, and behaviors of the individual (Kelman, 1974). The “differential order pattern” characteristic of Chinese farmer cooperatives (Ye and Wei, 2016) has contributed to the formation of strong relationships with higher trust and tightness, providing influence or favors to people within the network (Granovetter, 1973), further helping cooperative members to accept information and new things (Luo et al., 2011). For example, Zhang et al. (2021) showed that farmers can obtain favorable information through their personal relationship networks as a way to compensate for the narrow government information channels and the weak information interpretation abilities of farmers. Then, for the average member of a farmer cooperative in a strong relational network, the strength of the relationship with other members determines its network position, which further affects access to resources and individual benefits. Therefore, the cooperative social impact of the continuous use of new agricultural technology for the members of the cooperative is the extent to which the continuous use of the technology is influenced by the president of the cooperative, the members of the board of directors, and other members of the cooperative.

Based on the research situation of Chinese farmer cooperatives, Chen and Liu (2018) stated that the sense of organizational belonging is an important factor affecting the enthusiasm and initiative of cooperative members. In addition, since farmer cooperatives have the dual attributes of enterprise and community, the innovation ability of the directors becomes an important factor affecting the development and performance of cooperatives (Hu, 2013). Therefore, this study shows that the social impact of cooperatives can be considered from the following two dimensions: an organizational sense of belonging and the innovation ability of the director.

There are numerous related studies. Tortoriello et al. (2012) found that information generated through regular interactions and exchanges can facilitate information sharing and resource exchange, thus helping to cope with the impacts of environmental changes and various uncertainties. Based on the perspective of relational networks among farmers, Yu et al. (2019) found that strong relationships can deepen trust among farmers, promote the formation of mutual cooperation and information-sharing mechanisms, and facilitate the adoption of new technologies. Finally, in their study of the participation of farmer cooperatives in agricultural technology extension, Zheng et al. (2017) found that strong relationships in cooperatives significantly and positively influenced the agricultural technology extension performance within cooperatives.

In summary, it is clear from the existing studies that strong relationships play a positive role in improving firm performance. Therefore, this study incorporates the unique attributes of farmer cooperatives and infers that cooperative social impact has a positive and significant effect on the intention of cooperative members to consistently use new agricultural technologies, with the following hypothesis:

H3: The cooperative social impact will significantly and positively influence the continuous use intention toward new technologies in agriculture.

### 2.4. Policy support and continuous use intention

According to the definition of the Organization for Economic Co-operation and Development (OECD), agricultural support policy is the support, subsidies, assistance, and aid given to farmers or agriculture in general by the government to increase the income of farmers or reduce their costs. For Chinese farmer cooperatives, their dual economic and social attributes dictate that they can play an active role in areas where the market allocation of resources is uneven or ineffective. However, farmer cooperatives are at a disadvantage in competition with other profit-making enterprises and need corresponding support from the government to improve the quality of agricultural products, increase the income of farmers, and promote the benign development of cooperatives. For example, Xu (2014) analyzed the characteristics of farmer cooperatives as a policy tool for the government to achieve “state intentions” from the perspective of empowerment theory and found that they determine the inevitability of government policy support. At the same time, policy support is also the “first driving force” for the development of cooperatives. Therefore, in this study, policy support refers to the relevant policies and technical training organized by the government to support and guide the members of farmer cooperatives in using new agricultural technologies in the agricultural production process.

Based on the research situation of Chinese farmer cooperatives, Luo et al. (2022) stated that technical training can play an important role in promoting the application of new agricultural technologies. In addition, since the Chinese farmer cooperatives follow the development path of “government support and farmer initiative,” the continuity of government support policies is one of the considerations for promoting the application of new agricultural technologies (Liu, 2016). Therefore, this study suggests that policy support can be considered from the following two dimensions: technical training and policy stability.

There are numerous relevant studies on policy support. For example, Tate (2010) found through a survey of farmers in Shropshire, UK, that changes in European agricultural and environmental policies between 1997 and 2009 influenced the entrepreneurial behavior of farmers. Zhu and Kang (2013) found that policy support can positively influence the entrepreneurial intentions of farmers when they explored the logical relationship between the financial environment, policy support, and entrepreneurial intentions of farmers. Finally, in their study of the factors influencing the performance of cooperatives, Deng et al. (2021) found through empirical analysis that external support policies significantly and positively affected the performance of cooperatives.

In summary, policy support could have a positive effect on the intention to sustain the use of new agricultural technologies. In other words, a series of supporting policies formulated by the government, as well as technical training organized under the leadership of the government, would dispel the concerns of cooperative members about the continuous use of new agricultural technologies, enhance confidence in their continuous use, and have a positive impact on the continuous use intention toward new agricultural technologies. Therefore, this study infers that policy support has a positive and significant effect on the intention to sustain the use of new agricultural technologies and proposes the following hypothesis:

H4: Policy support will significantly and positively influence the continuous use intention toward new technologies in agriculture.

### 2.5. Moderating effects

In the UTAUT model proposed by Venkatesh et al. (2003), four moderating variables, namely, gender, age, experience, and voluntariness of use, indicate that individual characteristics and social factors are key factors to be considered for individual heterogeneity. Therefore, based on the UTAUT model, this study introduced the organizational model of farmer cooperatives as a moderating variable by combining the organizational characteristics of farmer cooperatives to explore the moderating effect on the intention to use new technologies consistently. This study argues that the organizational model of farmer cooperatives mainly refers to the different industrialized organizational and business models formed by vertical collaboration and vertical integration, among others, between farmer cooperatives and other agricultural business entities. In studying the mechanism of influence between government support and farmer cooperative regularization, Lin and Wu (2018) found that there is a moderating effect of the farmer cooperative organizational model in the relationship between government support and cooperative regularization. Moreover, Lu et al. (2017) showed that the demand for emerging agricultural technologies, as well as the rate of updating, varies among different models of farmer cooperatives.

Based on this, this study attempted to test whether the moderating effects of different organizational models of farmer cooperatives exist between performance expectations, effort expectations, cooperative social impact, and the intention to use new agricultural technologies consistently.

H5: The organizational model of farmer cooperatives will moderate the relationship between the performance expectations of members and continuous use intention toward new technologies in agriculture.
H6: The organizational model of farmer cooperatives will moderate the relationship between the effort expectations of members and continuous use intention toward new technologies in agriculture.
H7: The organizational model of farmer cooperatives will moderate the relationship between the social impacts of members and continuous use intention toward new technologies in agriculture.

**Figure 1:**
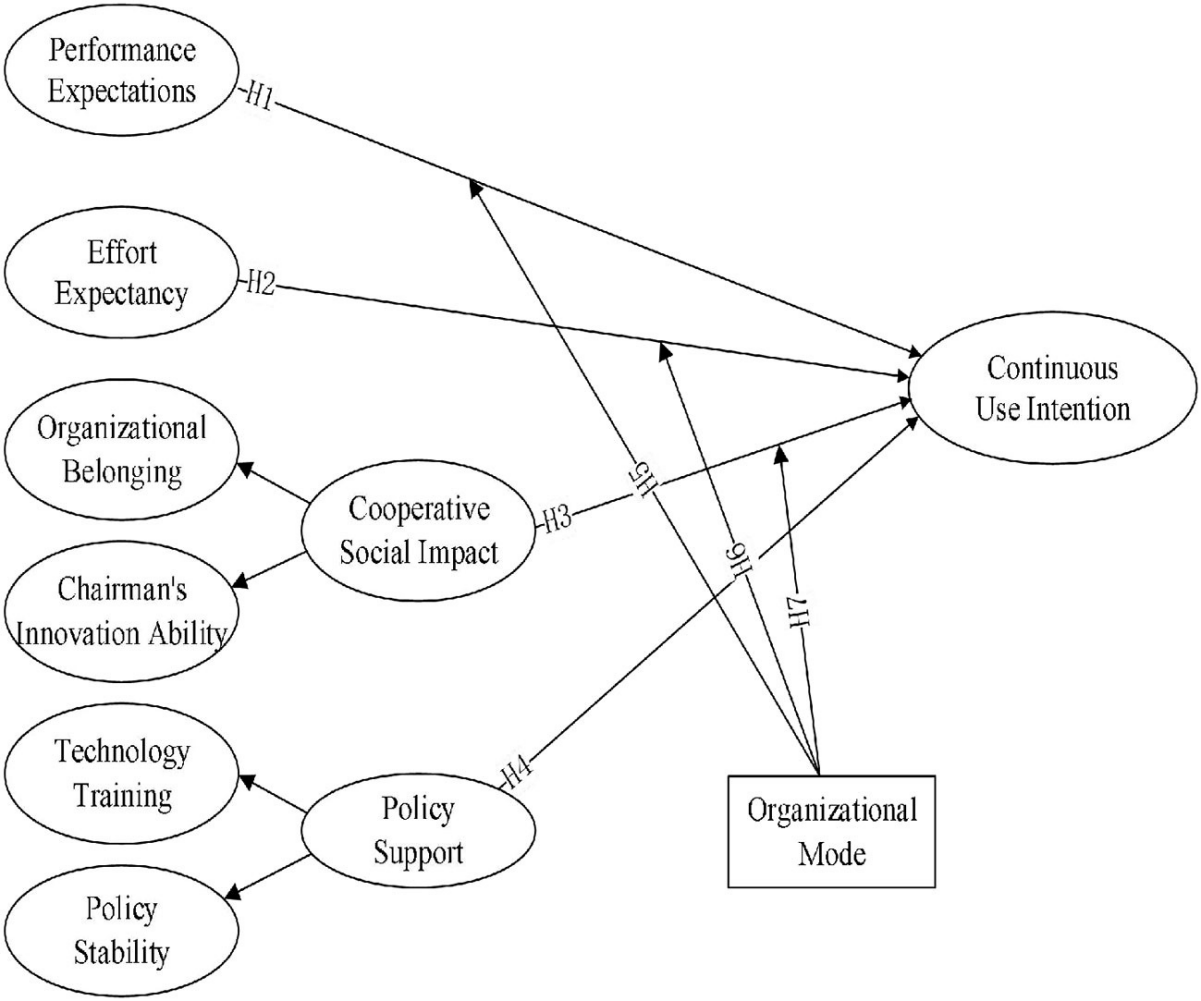
Theoretical framework.

In summary, this study proposes a research structure as shown in Figure 1.

## 3. Research design

### 3.1. Research subjects and data collection

In this study, data were collected using a web-based electronic questionnaire, and then, the research model of this study was tested empirically. This study was conducted to examine the intention of farmer cooperative members to continue using new technologies in agriculture, and therefore, the study population was set to include members of various types of farmer cooperatives within mainland China. To ensure that the questionnaire is logical, standardized, and organized, the standard “translation-back translation” method was used (Xia and Andrews, 2015). Seven scholars with experience in related fields were invited to complete the content revision to fit the research context of farmer cooperatives. An anonymous questionnaire was used to solicit respondents for this study to avoid issues of invasion of personal privacy. The questionnaires were distributed from 1 April 2021 to 31 May 2021, and a total of 509 questionnaires were collected. After removing invalid questionnaires, a total of 401 valid questionnaires were collected. The ratio of the sample size to sample question items in this study is 11.79, which satisfies the sample size requirement for empirical studies (Bagozzi and Yi, 2012).

### 3.2. Latent variable measurement

The questionnaire items were scored on a seven-point Likert scale ranging from strongly disagree (1), disagree (2), somewhat disagree (3), average (4), somewhat agree (5), agree (6), and strongly agree (7), with higher scores representing higher levels of agreement with the research variables. After the questionnaire items were designed, experts and scholars were invited to review the items and give their opinions. The design of each structure questionnaire is described as follows.

#### 3.2.1. Performance expectations

In this study, the construct of performance expectations refers to the study by Zheng et al. (2020). The study defined performance expectations as the extent to which business users perceive that the adoption of new information technology will enhance business performance. This study adopts two items from its questionnaires. The original questions for the “performance expectations” section, such as “Using IIP will improve our productivity” and “Using IIP will improve our product quality,” were revised by referring to the continuous use of new agricultural technologies. This study also adopted research by Tian and Yan (2020) on performance expectations by choosing the following two questions: “I think adopting e-commerce can reduce transaction costs” and “I think adopting e-commerce can increase revenue.” Finally, the question “Blockchain technology increases my productivity” was modified with reference to Lobel and Phuong (2021) for exploring the performance expectations after the adoption of blockchain technology. There were a total of five items in the construct of performance expectations.

#### 3.2.2. Effort expectations

The study by Ding and Xu (2019) was referred to for the construct of effort expectations of new professional farmers using the S&T information service system. This study revised the items “I think the scientific and technological information obtained through S&T information service is easy to understand and master” and “I think I can easily apply the information and knowledge provided by S&T information service in my work.” In addition, the study of Bao (2017) was referred to by adopting “I think mobile learning devices are easy to use” and “I think I can easily apply information and knowledge provided by technology information services in my work.” Finally, the study of Wissal et al. (2021) was referred to as “Interaction with IoT products for healthcare is easy for me.” There was a total of 5 questions in this construct.

In this study, the variables of cooperative social impact were mainly measured by the sense of organizational belonging and the innovation ability of the director.

#### 3.2.3. Organizational belonging

This study referred to the following three items of the Social Capital Measurement Scale of Gui and Huang (2008): “I feel at home in my neighborhood,” “I like my neighborhood,” and “I am proud to tell others where I live.” This study also referred to the questions in Mark et al. (2019) “I felt accepted by my co-workers” and “I received good support from my co-workers,” regarding the work relationship scale in companies. There was a total of five questions in this construct.

#### 3.2.4. Innovation ability of the chairman

This study referred to the innovation ability of the measurement scale of chairman of the study by Zeng and Li (2016), including “you often have new ideas about cooperative system construction and product management,” “you adopt new technologies and methods before your competitors,” “you make bold decisions in the face of uncertainty,” and “you prefer high-risk and high-reward projects.” Furthermore, the question of Guo et al. (2019), “You are able to judge various changes in the market and develop a response plan,” was adopted. There was a total of five questions in this construct.

The variables of policy support in this study were mainly measured by technical training and policy stability.

#### 3.2.5. Technical training

In this study, we refer to the technical training measurement scale of the study by Ge et al. (2022), in which the following two questions were asked: “Cooperative members have many training opportunities” and “Training content is specific, practical and rich.” In addition, according to the study by Zhu and Liu (2013), “The training on the use of Sakai was comprehensive,” “The training improved the understanding of Sakai,” “The training improved the proficiency of operation,” and “The trainers were experienced and proficient in responding to questions” were revised to a total of five questions.

#### 3.2.6. Policy stability

In this study, the items “the government has always paid high attention to technology entrepreneurship policies,” “the technology entrepreneurship policies introduced in different time periods have a strong consistency,” and “the government constantly adjusts the technology entrepreneurship policies according to the changes in the situation” were modified by referring to the item in the scale of policy continuity measurement by Peng et al. (2017), and a total of four questions were developed.

#### 3.2.7. Continuous use intention

This study referred to the measurement scale “I would recommend other companies to use IoT technology” in the study by Dong and Hu (2020) on the willingness of logistics companies to use IoT technology consistently. Moreover, items of the study by Maroua (2018), “I intend to continue using the Blackboard for knowledge gathering,” “I intend to continue using the Blackboard for knowledge construction,” “I intend to continue using the Blackboard for knowledge sharing,” and “Overall, I intend to continue using the Blackboard” were modified as questionnaire items. There was a total of 5 questions in this construct.

### 3.3. Data analysis

In this study, the results of the sample data were analyzed through three stages, namely, descriptive statistical analysis, measurement model validation, and structural equation modeling analysis. First, the descriptive statistical analysis of SPSS mainly completed the consistency analysis of facet measurement items. Second, based on the findings of Anderson and Gerbing (1988), this study completed the validation of the measurement model through reliability and discriminant validity. Confirmatory factor analysis (CFA) was used to confirm the reliability of the items and the internal consistency of the measurement using the composite reliability (CR) and average variance extracted (AVE). In addition, discriminant validity was tested by comparing the square root of the AVE value and correlation coefficients. Finally, based on the research model, path analysis and moderating effect analysis were performed on the structural equation model.

## 4. Results

### 4.1. Descriptive statistics

From the statistics of each question item (Table 1), the means of the seven latent variables in this study lie between 4.974 and 5.127, all of which are greater than the median, indicating that all seven latent variables are in the middle to upper range. The standard deviation was between 1.474 and 1.694, indicating a good consistency in the respondents'evaluation of the 34 measure items.

**Table 1:** Question item analysis.

**Construct**	**Item**	**Mean**	**Standard deviation**
Performance expectations (PE)	PE1	5.000	1.621
	PE2	4.959	1.651
	PE3	5.005	1.651
	PE4	5.008	1.613
	PE5	4.951	1.630
Effort expectations (EE)	EE1	5.022	1.576
	EE2	5.016	1.609
	EE3	5.033	1.573
	EE4	5.049	1.572
	EE5	4.992	1.642
Organizational belonging (OB)	OB1	5.000	1.585
	OB2	4.978	1.592
	OB3	5.027	1.578
	OB4	5.003	1.600
	OB5	5.024	1.568
Chairman's innovation ability (CIA)	CIA1	4.973	1.586
	CIA2	5.005	1.534
	CIA3	5.043	1.581
	CIA4	5.092	1.539
	CIA5	5.008	1.572
Technology training (TT)	TT1	4.954	1.633
	TT2	4.997	1.694
	TT3	5.054	1.669
	TT4	4.965	1.689
	TT5	4.967	1.673
Policy stability (PS)	PS1	5.076	1.555
	PS2	5.011	1.515
	PS3	5.111	1.501
	PS4	5.106	1.474
Continuous use intention (CUI)	CUI1	5.084	1.449
	CUI2	5.152	1.557
	CUI3	5.117	1.527
	CUI4	5.090	1.527
	CUI5	5.090	1.545

### 4.2. Measurement model verification

#### 4.2.1. Reliability analysis

Fornell and Larker (1981) suggested that a critical value of 0.7 for the combined reliability value indicates the internal consistency of the measured question items for each construct. Therefore, in this study, confirmatory factor analysis of latent variables was performed to obtain standardized factor loadings (SFL), Cronbach's alpha, rho A, composite reliability (CR), and average variance extracted (AVE), and the results are shown in Table 2 for reliability analysis and the next step of validity analysis.

**Table 2:** Results of the confirmatory factor analysis.

**Latent variable**	**Item**	**SFL**	**Cronbach's alpha**	**Rho A**	**CR**	**AVE**
Performance expectations (PE)	PE1	0.913	0.956	0.956	0.966	0.851
	PE2	0.924				
	PE3	0.927				
	PE4	0.922				
	PE5	0.926				
Effort expectations (EE)	EE1	0.904	0.950	0.951	0.962	0.834
	EE2	0.922				
	EE3	0.907				
	EE4	0.910				
	EE5	0.923				
Continuous use intention (CUI)	CUI1	0.883	0.940	0.941	0.955	0.808
	CUI2	0.911				
	CUI3	0.894				
	CUI4	0.891				
	CUI5	0.904				
Organizational belonging (OB)	OB1	0.913	0.954	0.954	0.965	0.846
	OB2	0.918				
	OB3	0.924				
	OB4	0.924				
	OB5	0.919				
Chairman's innovation ability (CIA)	CIA1	0.912	0.949	0.949	0.960	0.829
	CIA2	0.910				
	CIA3	0.924				
	CIA4	0.902				
	CIA5	0.905				
Technology training (TT)	TT1	0.928	0.959	0.959	0.967	0.860
	TT2	0.927				
	TT3	0.926				
	TT4	0.928				
	TT5	0.928				
Policy stability (PS)	PS1	0.919	0.934	0.935	0.953	0.834
	PS2	0.923				
	PS3	0.905				
	PS4	0.907				

#### 4.2.2. Discriminant validity

This study used the more rigorous average variance extracted (AVE) method to examine discriminant validity. Straub et al. (2004) suggested that the square root of the average variance extracted value (AVE) for each construct needs to be greater than the value of the correlation coefficient between the paired constructs. This indicates the discriminant validity among the constructs, as shown in Table 3. The square root value of AVE corresponding to each variable was greater than the correlation coefficient with other variables, indicating that the measurement scale as a whole has good discriminant validity.

**Table 3:** Results of discriminant validity tests.

	**Effort expectations**	**Technology training**	**Continuous use intention**	**Policy stability**	**Chairman's innovation ability**	**Organizational belonging**	**Performance expectations**
Effort expectations	**0.913**						
Technology training	0.535	**0.927**					
Continuous use intention	0.635	0.639	**0.899**				
Policy stability	0.606	0.620	0.707	**0.913**			
Chairman's innovation ability	0.536	0.556	0.646	0.608	**0.911**		
Organizational belonging	0.554	0.523	0.624	0.604	0.535	**0.920**	
Performance expectations	0.515	0.544	0.638	0.610	0.559	0.544	**0.923**

The bold diagonal line represents the square root value of AVE for each construct, while the lower triangle shows the Pearson correlation coefficient value between constructs.

### 4.3. Structural equation modeling

#### 4.3.1. Model goodness-of-fit test

In the structural equation modeling validation process, there is the problem that the model can explain the changes in the endogenous latent variables, but cannot fit the data well. Therefore, the goodness-of-fit (GOF) test was used in this study to determine the fitness of the model. According to Wetzels et al. (2009), the model fit is weak when the GOF is < 0.25, moderate when it is between 0.25 and 0.36, and good when it is >0.36. In the present study, the criteria of the fit requirements were met, indicating that the model fit was good and the analysis of the structural model could be performed.

#### 4.3.2. Path analysis

Table 4 shows the path coefficients, including performance expectations (*t*-value = 4.182 > 1.96, *p*-value < 0.001), effort expectations (*t*-value = 3.897 > 1.96, *p*-value < 0.001), policy support (*t*-value = 6.272 > 1.96, *p*-value < 0.001), and cooperative social impact (*t*-value = 5.309 > 1.96, *p*-value < 0.001), which all significantly affect continuous use intention. The findings support the research hypotheses of this model, with performance expectations, effort expectations, cooperative social impact, and policy support having 66.0% of the explanatory power to explain continuous use intention.

**Table 4:** Path analysis.

**Hypothesis**	**Original sample (O)**	**Sample mean (M)**	**Standard deviation (STDEV)**	***t*-value (|O/STDEV|)**	***p*-value**	***R* ^2^**
Performance expectations → Continuous use intention	0.167	0.168	0.040	4.182	0.000^***^	0.660
Effort expectations → Continuous use intention	0.170	0.172	0.044	3.897	0.000^***^	
Cooperative social impact → Continuous use intention	0.276	0.271	0.052	5.309	0.000^***^	
Policy support → Continuous use intention	0.331	0.330	0.053	6.272	0.000^***^	

^***^p < 0.001.

#### 4.3.3. Moderating effects

In this study, the moderating effect analysis was completed using the Smart PLS 3.0 software. The results of the analysis in Table 5 show that, on the path of performance expectations on the continuous use intention, hypothesis H5 does not hold. On the path of effort expectations on the continuous use intention, hypothesis H6 is partially valid. On the path of cooperative social impact on the continuous use intention, hypothesis H7 is partially valid.

**Table 5:** Analysis of the moderating effect of the organizational model of farmer cooperatives.

	**PE → CUI**	**EE → CUI**	**CSI → CUI**
Path coefficients-diff (village elite leadership type vs. others)	0.312	0.613	0.938
Path coefficients-diff (village elite leadership type vs. government department driven)	0.016	−0.610	0.359
Path coefficients-diff(government department driven vs. leading enterprises driven type)	−0.176	0.040	0.018
Path coefficients-diff (others vs. government department driven)	−0.296	−1.223	−0.579
Path coefficients-diff (others vs. leading enterprises driven type)	−0.488	−0.573	−0.919
Path coefficients-diff (government department driven vs. leading enterprises driven type)	−0.192	0.650	−0.341
*t*-value (|village elite leadership type vs. others|)	1.007	1.228	1.887
*t*-value (|village elite leadership type vs. government department driven|)	0.149	5.121^*^	2.329^*^
*t*-value (|village elite leadership type vs. leading enterprises driven type|)	1.026	0.483	0.117
*t*-value (|others vs. government department driven |)	0.648	1.611	0.896
*t*-value (|others vs. leading enterprises driven type |)	0.863	1.054	1.619
*t*-value (|government department driven vs. leading enterprises driven type |)	0.753	5.096^*^	1.819
*p*-value (village elite leadership type vs. others)	0.315	0.221	0.061
*p*-value (village elite leadership type vs. government department driven)	0.881	0.000	0.021
*p*-value (village elite leadership type vs. leading enterprises driven type)	0.306	0.629	0.907
*p*-value (others vs. government department driven)	0.519	0.111	0.373
*p*-value (others vs. leading enterprises driven type)	0.390	0.294	0.108
*p*-value (government department driven vs. leading enterprises driven type)	0.453	0.000	0.070

PE, performance expectations; EE, effort expectations; CSI, cooperative social impact; CUI, continuous use intention.

^*^p < 0.05.

## 5. Conclusion and discussion

### 5.1. Academic contributions

This study attempts to explore the factors influencing the continuous use intention toward new agricultural technologies from the perspective of the members of farmer cooperatives in China based on the UTAUT model and the social impact and policy support dimensions of cooperatives. The results are obtained as follows.

#### 5.1.1. Effect of performance expectations and effort expectations on continuous use intention

The results of the study showed that performance expectations have a significant positive effect on the continuous use intention toward agricultural technologies (β = 0.167, *p* < 0.001). In other words, the utility of the new agricultural technology itself, as well as the psychological expectations of the members of farmer cooperatives about the performance it brings, such as in terms of agricultural production efficiency and profitability, can influence the continuous use intention of cooperative members. The research results are consistent with the findings of Chen et al. (2017) and Ding and Xu (2019).

The results of the study showed that effort expectations have a significant positive effect on the intention to use new agricultural technologies consistently (β = 0.170, *p* < 0.001). It shows that the application of new agricultural technologies in the agricultural production process has certain requirements for the use of various terminal devices and platform software. The simplicity of the operation, the friendliness of the system interface, and the ease of reading and understanding agricultural information will affect the intention of cooperative members to use new agricultural technologies continuously. However, as new agricultural technologies are promoted and technical training is intensified, cooperative members learn and master new agricultural technologies faster, which makes it less difficult to use new agricultural technologies. They are more likely to use the system again, so the continuous use intention is strengthened accordingly. The findings are consistent with the view expressed by Wang (2014).

#### 5.1.2. Cooperative social intention on continuous use intention

The results of the study showed that cooperative social impact has a significant positive effect on the continuous use intention toward new agricultural technologies (β = 0.276, *p* < 0.001). In other words, ordinary members who are within the relational network within farmer cooperatives are influenced by the recommended attitudes of other members within the cooperative in the decision-making process for the continuous use of new agricultural technologies, which is consistent with the findings of Guo et al. (2019). At the same time, to promote the benign development of farmer cooperatives, innovative directors are prone to promote and use new agricultural technologies within the cooperative and influence the willingness of members to continue using them through the “competent person effect,” a finding consistent with that of Ye and Chen (2019).

The application of relational embedding theory to the study of the continuous use intention of farmer cooperative members toward the new agricultural technologies can explain the mechanism of the influence of network relationships within the cooperative on their willingness to use consistently. Ordinary farmers who join farmer cooperatives have their agricultural production and operation activities deeply embedded in the strong relationship network of farmer cooperatives. Therefore, in the decision-making process of the continuous use of new agricultural technologies, ordinary members not only judge through their own perceptions of the use of new agricultural technologies but also focus more on obtaining implicit knowledge of the utility of the use of new agricultural technologies through the strong relationship network to help them make the right choice to reduce uncertainty.

In particular, the innovation demonstrated by board chairs, who are at the nodes of strong relational networks of farmer cooperatives, led to the development of cooperatives. Through demonstration and leadership in the application of new agricultural technologies and information sharing, the chairpersons of farmer cooperatives are conducive to enhancing the confidence of ordinary members of cooperatives in the continued use of new agricultural technologies.

#### 5.1.3. Impact of policy support on continuous use intention

The results of the study showed that policy support has a significant positive effect on the continuous use intention toward new agricultural technologies (β = 0.331, *p* < 0.001). In other words, when new agricultural technologies are used in agricultural production and operation, members of farmer cooperatives perceive the intensity of support from the supporting policies of the government and the effectiveness of the various types of training organized, which dispels their cost concerns about the continuous use of new agricultural technologies and thus promotes the benefits of cooperative members. The results are consistent with the findings of Deng et al. (2021).

The application of political embedding theory to the study of the willingness of farmer cooperative members to use new agricultural technologies consistently can explain the mechanism of the effect of policy support on their continuous use intention toward new agricultural technologies. Based on the perspective of political embeddedness, the economic behavior of farmer cooperatives, as special economic subjects, is influenced by the political environment, power structure, and industrial policies in the process of interaction with government departments. Therefore, farmer cooperatives, which are formed by the union of scattered small farmers, can obtain government policy support through political embedding. It helps cooperatives obtain various resources, including agricultural projects and policy preferences, to promote the benign development of cooperatives. Since the organizational model of farmer cooperatives, in addition to being led by village elites, also includes models such as leading enterprises and government departments, while gaining legitimate social recognition, farmer cooperatives led by leading enterprises and government departments need to actively integrate into the village society to overcome “externalities” and play a role in promoting the use of new agricultural technologies throughout the village by leveraging the weak “government-public” duality among ordinary farmers (Zhao, 2019).

The empirical study found that the four main variables, namely, performance expectation, effort expectation, cooperative social influence, and policy support, have the following strengths on the intention to continue using them: policy support (0.331) > cooperative social influence (0.276) > effort expectation (0.170) > performance expectation (0.167). The results indicate that the highest intensity of influence was found for policy support, reflecting the dominant role of policy support on the willingness of cooperative members to use new agricultural technologies consistently. This is consistent with the view expressed by Xu (2014) that “policy support is the ‘first driving force' of cooperative development.” The strength of the impact of cooperative social influence ranked second, reflecting that cooperative social influence plays a facilitating role in the willingness of cooperative members to use the AI system consistently, which is consistent with the findings by Tan et al. (2017).

#### 5.1.4. Moderating effects

In this study, hypothesis H5 was not supported, and both H6 and H7 were partially supported. The empirical study found that the organizational model of farmer cooperatives did not have a moderating effect on the path of performance expectations on continuous use intention. Hypothesis H5 does not hold. A possible explanation is that members of farmer cooperatives in the same organizational model have homogeneity of property rights structure, surplus distribution, and other systems so that the expectation of returns of cooperative members does not influence their continuous use intention toward new agricultural technologies.

The organizational model of farmer cooperatives possesses a partial moderating effect on the path of effort expectations on continuous use intention, i.e., hypothesis H6 partially holds. A possible explanation is that farmer cooperatives with different organizational models have different levels of demand for new agricultural technologies and different rates of updating (Lin and Wu, 2018), and the intensity of the introduction of new agricultural technologies is as follows: “Leading enterprises driven type” > “Government department driven” > “Village elite leadership type” > “other” types. Then, for cooperative members, the cost of time and effort to master new agricultural technologies varies among farmer cooperatives in different organizational models due to the various introduction and update rates of new agricultural technologies having a significant impact on their continuous use intention.

The organizational model of farmer cooperatives possesses a partial moderating effect on the path of the cooperative social impact on continuous use intention, i.e., hypothesis H7 partially holds. A possible explanation is that the homogeneous differences possessed by the internal network relations of farmer cooperatives with different organizational models affect the dissemination of tacit knowledge (Granovetter, 1973; Lu et al., 2017). Then, it is easier for cooperative members to acquire tacit knowledge about new agricultural technologies to help them cope with economic risks and uncertainty shocks when making decisions about the continuous use of new agricultural technologies, such as the “Village elite leadership type.” The inclusion of enterprise representatives or government agents in the “Leading enterprises driven” and “Government department driven” organizational structures not only enhances the accessibility of resources for cooperative members but also reduces the dissemination of tacit knowledge and leads to a reduction in the homogeneity of internal network relationships, which is characterized by “weak relationships” and affects the intention toward cooperative members to continue using them.

### 5.2. Management insights

Based on the results of this study, the following recommendations are made to promote relevant policies to enhance the continuous use intention of cooperative members toward the new agricultural technologies.

First, the agricultural sector of government can strengthen policy guidance. The policy support component of the study shows that policy support is the most critical influencing factor for the intention of farmer cooperative members toward the continuous use of new agricultural technologies. Therefore, it is recommended that government departments effectively promote the implementation of new agricultural technologies in the field of agricultural production by enhancing the support of relevant policies, increasing investment in education and training, and ensuring the continuity and stability of supporting policies.

Second, farmer cooperatives focus on the training and introduction of talents. This study found that the rational allocation of talent laddering in agricultural cooperatives has an important role in promoting the benign development of cooperatives. Therefore, it is highly recommended that farmer cooperatives should combine their realities and strengthen the capacity cultivation of their directors through short-term study tours, training visits, forums, and exchanges. Moreover, they should focus on cooperative members and young farmers and on the internal excavation and cultivation of reserve talents through the cooperative system and relevant professional learning. Finally, they should broaden the channels to introduce talents, adhere to the policy of introducing as needed and flexibly, and hire professional managers with rich management experience, profound technical knowledge, and strong competitive consciousness through contractual constraints, equity incentives, and a combination of full-time and part-time jobs to realize the benign development of professional cooperatives of farmers.

Third, farmer cooperatives strengthen internal governance. The study of the social impact component of cooperatives shows that the sense of organizational belonging of cooperative members plays a helpful role in strengthening intra-cooperative network relationships and the dissemination of tacit knowledge about new agricultural technologies. Therefore, it is suggested that farmer cooperatives cultivate the sense of ownership of cooperative members, strengthen the strength of internal networks, enhance their sense of organizational belonging, and promote the use of new agricultural technologies by optimizing the existing internal management system, establishing an information communication mechanism and building a cooperative culture. Therefore, it is suggested that farmer cooperatives cultivate the sense of ownership of cooperative members, strengthen the internal networks, enhance their sense of organizational belonging, and promote the use of new agricultural technologies by optimizing the existing internal management system, establishing an information communication mechanism, and building a cooperative culture.

### 5.3. Research limitations and future developments

This study mainly focused on Chinese farmer cooperative members and collected sample data through a questionnaire survey. The sample data are cross-sectional and do not reflect the dynamics of the variables. The causal explanatory power of the mechanisms that lead to the influence of the continuous use intention toward the new agricultural technologies is insufficient, and future studies may further consider the use of longitudinal studies for validation. In addition, this study constructs a theoretical model from the perspective of farmer cooperative members to study the factors influencing continuous use intention. It does not consider factors such as satisfaction and performance, and future studies, as needed, can include other factors into the model to be studied.

## Data availability statement

The raw data supporting the conclusions of this article will be made available by the authors, without undue reservation.

## Author contributions

FJ’s contribution includes ideas, formulation of overarching research goals and aims, and writing. RZ’s contribution includes data collection and writing original draft preparation. JL’s contribution includes software operation and reviewing. All authors contributed to the article and approved the submitted version.
